# Supplementary figures and images for: Expansion of the HSFY gene family in pig lineages: HSFY expansion in suids
Source: BMC Genomics. 2015 Jun 9;16(1):442. doi: 10.1186/s12864-015-1650-x (PMC4460688; doi:10.1186/s12864-015-1650-x)

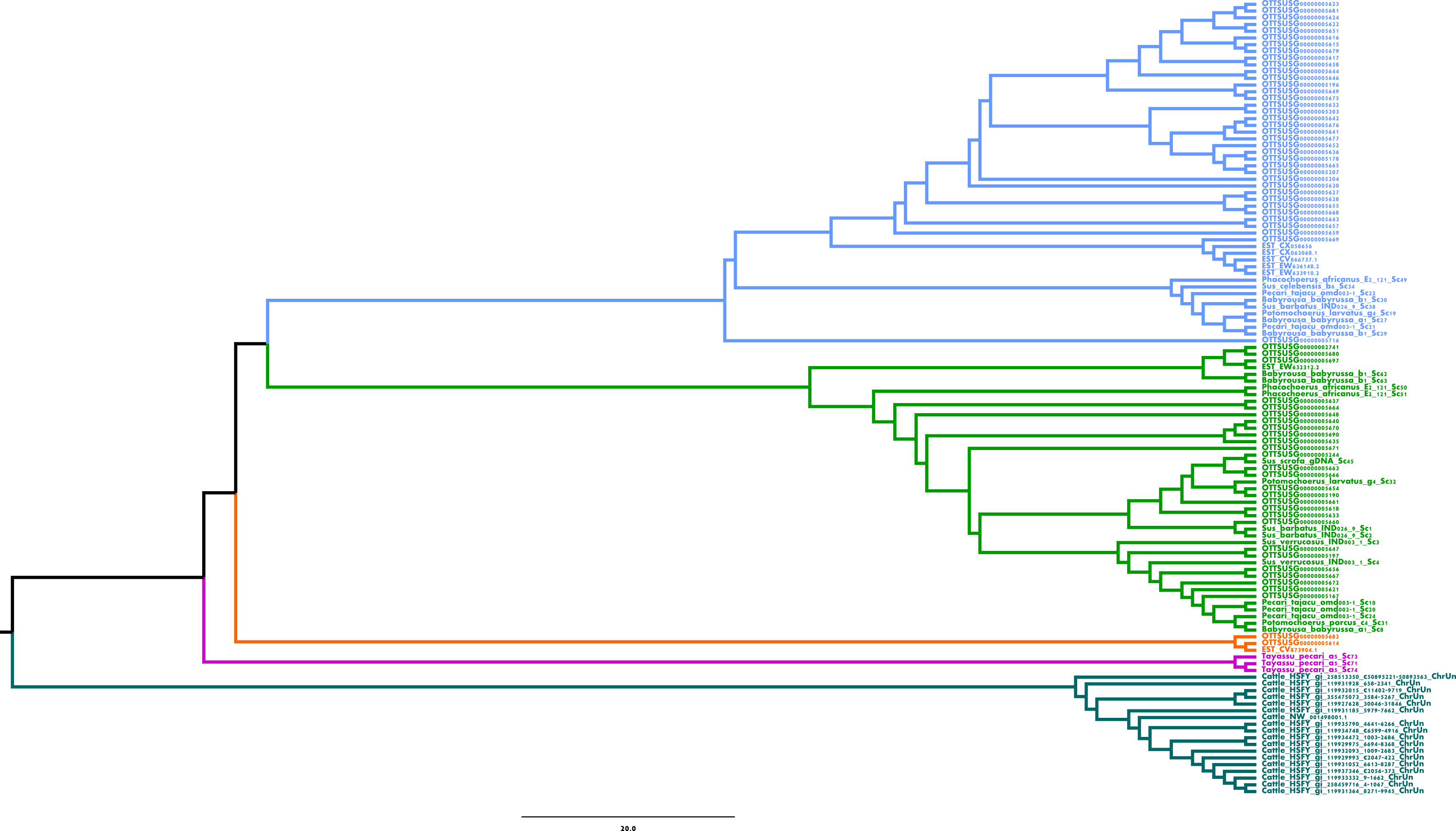

Supplement: Additional file 2: Figure S1. — Tree including cattle HSFY and pig EST sequences. HSFY sequences aligned as described in Fig. 3, with the inclusion of cattle HSFY sequences and pig HSFY ESTs. The clustering shows that the ESTs are almost all associated with the short form of HSFY, and that the cattle sequences are distinct from all suid copies, reflecting their independent amplification. [file 12864_2015_1650_MOESM2_ESM.png]
